# Efficacy and safety of immunotherapy for head and neck squamous cell carcinoma: a meta-analysis of randomized clinical trials

**DOI:** 10.3389/fonc.2024.1489451

**Published:** 2025-01-09

**Authors:** Cailing Jiang, Shuqin Wang, Lijun Zhu

**Affiliations:** Department of Oral and Maxillofacial Surgery, Guangdong Provincial People’s Hospital, Guangdong Academy of Medical Sciences, Southern Medical University, Guangzhou, China

**Keywords:** immunotherapy, squamous cell carcinoma of head and neck (HNSCC), overall survival (OS), progression-free survival (PFS), treat-related adverse events (TRAEs)

## Abstract

**Background:**

Head and neck squamous cell carcinoma (HNSCC) is one of the most common types of cancer worldwide and immune checkpoint inhibitors have shown favorable therapeutic effects in recurrent or metastatic or locally advanced head and neck squamous cell carcinoma (R/M/LA HNSCC). However, the effects of immunotherapy in HNSCC are still inconsistent because of complicating factors. This meta-analysis tries to provide a more precise assessment of the efficacy and safety of this integrated approach in HNSCC.

**Methods:**

We conducted a systematic review and meta-analysis of randomized clinical trials according to Preferred Reporting Items for Systematic Reviews and Meta-analyses (PRISMA) reporting guidelines. The outcomes were overall survival (OS), progression-free survival (PFS), and treatment-related adverse events (TRAEs). A total of 8 out of 2445 articles were analyzed, including 5067 HNSCC patients, including 823 and 4244 patients with LA HNSCC and R/M HNSCC.

**Results:**

The combined data revealed that immunotherapy has an apparent difference in OS (HR 0.86 95% CI 0.77-0.98) compared with standard of care (Soc, like fluoropyrimidine, methotrexate, docetaxel, or cetuximab) but was equal with the other treatment in PFS (HR 1.08, 95% CI 0.85-1.37). Furthermore, the occurrence of grade 3 or higher adverse events related to the drugs was lower than systematic therapy (OR 0.35, 95% CI 0.17-0.73).

**Conclusions:**

The study has provided compelling evidence that immunotherapy is a significant benefit in OS for HNSCC patients, either R/M HNSCC or LA HNSCC, immunochemotherapy may benefit more for these patients, but double-agent immunotherapy showed no more benefit for R/M HNSCC patients.

**Systematic Review Registration:**

https://www.crd.york.ac.uk/, identifier CRD42023471570.

## 1 Introduction

Head and neck squamous cell carcinomas (HNSCC) are derived from the mucosal epithelium in the oral cavity, pharynx, and larynx and are the most common malignancies that arise in the head and neck (1). HNSCC is the sixth most common cancer worldwide, with 890,000 new cases and 450,000 deaths in 2018. The incidence of HNSCC continues to rise and is anticipated to increase by 30% (that is 1.08 million new cases annually) by 2030 (2). Due to the complex anatomy of the head and neck, more than 50% of the HNSCC patients were diagnosed in clinical stage III or IV, and the survival rate is only 40~50%. Besides, local recurrence or metastasis also leads to the poor prognosis of HNSCC (3). Multimodal treatments include surgery, radiotherapy, chemotherapy, and molecular-targeted therapy. Despite the continuous innovation of treatment methods, there are still problems such as insufficient efficacy and excessive toxicity (4).

Recent understanding of the role of immune dysfunction in HNSCC has quickly established immunotherapy (IMT) as a promising treatment avenue (5). The monoclonal antibodies (mAbs) anti-programmed death protein-1 (anti-PD-1) nivolumab and pembrolizumab are the first immune checkpoint inhibitors (ICIs) approved for the treatment of patients with recurrent or metastatic HNSCC (R/M HNSCC) (6, 7). Anti-programmed death ligand-1 (anti-PD-L1) checkpoint inhibitors such as durvalumab and avelumab have been approved for the treatment of patients with locally advanced or metastatic HNSCC under phase III clinical trials since 2017 (8). In particular, the PD-1 therapy effect is mediated by the binding with T lymphocytes resulting in a systemic effect, whereas the activity of anti-PD-L1 therapy is directed against the receptor expressed on tumor cells (9, 10). Immune checkpoint molecules cytotoxic T lymphocyte antigen 4 (CTLA-4) like tremelimumab and ipilimumab also have been used in HNSCC (11).

Unfortunately, the most optimum regime for HNSCC is still unclear. Many recommendations are based on single RCT results or anti-PD-1/PD-L1 pathway IMT meta-analysis. In this review, we appraised RCTs evaluating the efficacy and safety of immunotherapy in patients with HNSCC through a systematic review and meta-analysis. The activity of systemic treatment will be assessed through overall survival (OS), progression-free survival (PFS), overall response rate (ORR), duration of response (DoR) (12), and treatment-related adverse events (TRAEs).

## 2 Materials and methods

### 2.1 Search strategy and selection criteria

**Figure 1:**
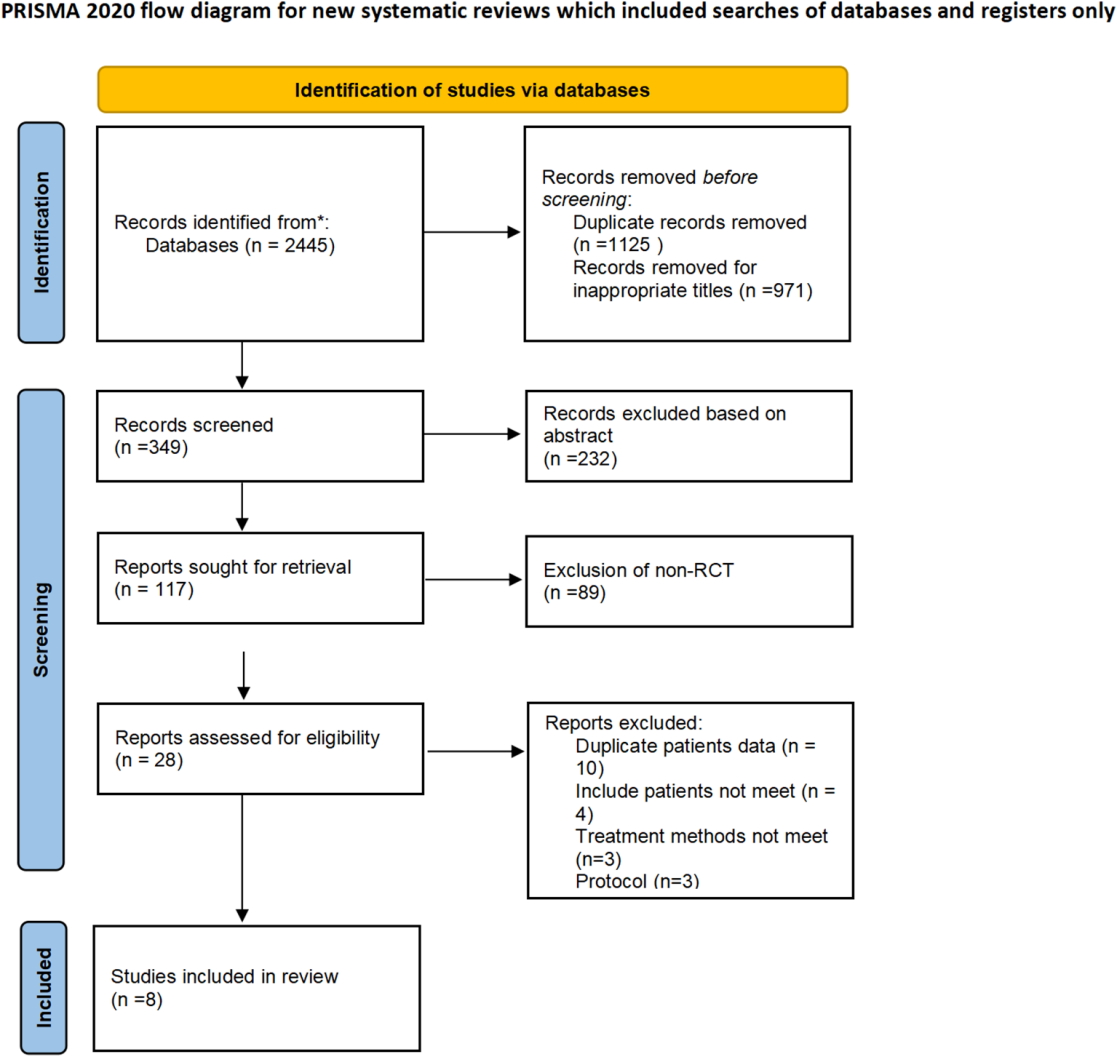
Flow diagram of trial selection. RCT: randomized clinical trials.

We performed a systematic literature review and meta-analysis of peer-reviewed journals published between 2004 to December 2023 from PubMed, Medline, Embase, and Web of Science. The search strategies used a combination of subject headings (e.g., MeSH in PubMed) and keywords such as head and neck squamous cell carcinoma, immunotherapy, and randomized clinical trials (RCTs). The English language only was applied to the search. Clinical trials were restricted to phase II and phase III RCTs (**Figure 1**).

Bibliographies of review articles and editorials were manually searched. The literature review process followed the Preferred Reporting Items for Systematic Reviews and Meta-Analyses (PRISMA) statement checklist and flowchart (13). Two authors (C.L Jiang and S.Q Wang) independently conducted the literature search, evaluated data from eligible studies, and data extraction, which were then checked by a third author (L.J Zhu). If a trial was reported by several publications, we included the most recent results.

Inclusion criteria were: ① patients with histologically confirmed HNSCC; ② patients received immunotherapy; ③ the study compared systemic therapy; ④ the study provided the hazard ratios (HRs) for OS or reported information to calculate these; ⑤ phase II~III, RCTs. Exclusion criteria were: ① nasopharyngeal cancer or esophageal carcinoma; ② trial in abstract forms or protocol report; ③ studies lacking relevant statistics.

### 2.2 Quality assessment and risk of bias

The selected studies were assessed with the Cochrane Collaboration’s risk of bias tool, which is used to assign a rating of low, unclear risk of bias, or high for the domains of selection, performance, detection, attrition, reporting, and others (**Supplementary Figure 1A**).

### 2.3 Data extraction and analysis

The following data were extracted from each article: trial identifier, author, publication year, journal, study design, intention-to-treat (ITT) population, age, gender, race, cancer stage, drug and dose, follow-up, OS, PFS, ORR, DoR, and TRAEs.

The primary endpoint was OS, secondary endpoints were PFS, ORR, DoR, and TRAEs. The OS, PFS, and DoR were expressed as HR for the meta-analysis, and ORR and TRAEs were reported as odds ratio (OR). If there were no (or all) events reported in both treatment groups, the study was excluded from the meta-analysis. A forest plot was constructed, including the overall effect, Cochran’s Q chi-square test, and I² statistics. The Cochran’s Q chi-square test and I² statistics were used to determine heterogeneity across the included trials, and I² values of 25%, 50%, and 75% were considered to indicate low, moderate, and high inconsistencies, respectively. A fixed effects model was used if the studies had low heterogeneity (P>0.1, I²<50%). Otherwise, a random-effect model was applied. All statistical analyses were conducted with Review Manager version 5.3. A significant result was indicated by a p-value of <0.05 for any measured outcomes.

## 3 Results

Study selection was conducted according to the PRISMA flowchart (**Figure 1**). The initial database query retrieved 1120 studies, where 28 full-text articles were screened for eligibility. Eight RCTs met the inclusion criteria and were included in the meta-analysis of randomized clinical trials (supplement data extraction). Characteristics of the included studies are described in **Table 1**. Seven studies were phase III (7, 14–19)and one study was phase II (20). A total of 4207 patients were included, most patients were male (81.54%), and the median age was 58.8 years. The median follow-up was 21.3 months. No trial had a low bias risk (**Supplementary Figure 1**).

**Table 1:** Characteristics of included trials.

Identifier	Author, year	Study phase	masking	Intervention model	Main inclusion criteria	Sample size	Outcome reported
NCT02105636	Ferris, R.L,2018	Phase 3	Open-label	Dual-arm	R/M HNSCC;	361	OS; PFS; ORR;
NCT02252042	Cohen, E.E.W,2019	Phase 3	Open-label	Dual-arm	R/M HNSCC;	495	OS; PFS
NCT02369874	Ferris, R.L,2020	Phase 3	Open-label	Three-arm	R/M HNSCC	736	OS; PFS, ORR
NCT02952586	Lee, N. Y. 2021	Phase 3	Double-blind	Dual-arm	LA HNSCC	692	PFS; TRAEs
NCT02741570	Haddad, R.I,2023	Phase 3	Double-blind	Dual-arm	R/M HNSCC	947	OS; PFS; ORR; DOR
NCT02358031	Harrington, K.J,2023	Phase 3	Open-label	Three-arm	R/M HNSCC	882	PFS; OS; ORR; DoR
NCT02551159	Psyrri, A,2023	Phase 3	Open-label	Three-arm	R/M HNSCC;	823	OS; PFS; ORR; DoR; TRAEs
NCT02707588	Tao, Y. 2023	Phase 2	Open-label	Dual-arm	LA HNSCC	131	PFS; OS

recurrent or metastatic (R/M); head-and-neck squamous cell carcinoma (HNSCC); locally advanced (LA); overall survival (OS); progression-free survival (PFS); objective response rate (ORR); duration of response (DoR); treatment-related adverse events (TRAEs)

**Figure 2:**
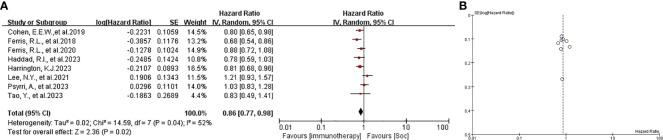
**(A)** forest plot OS analysis of immunotherapy in HNSCC. **(B)** funnel plot of survival benefits associated with immunotherapy versus SoC. OS, overall survival. SoC, standard of care. HNSCC, head and neck of squamous cell carcinoma.

**Figure 3:**
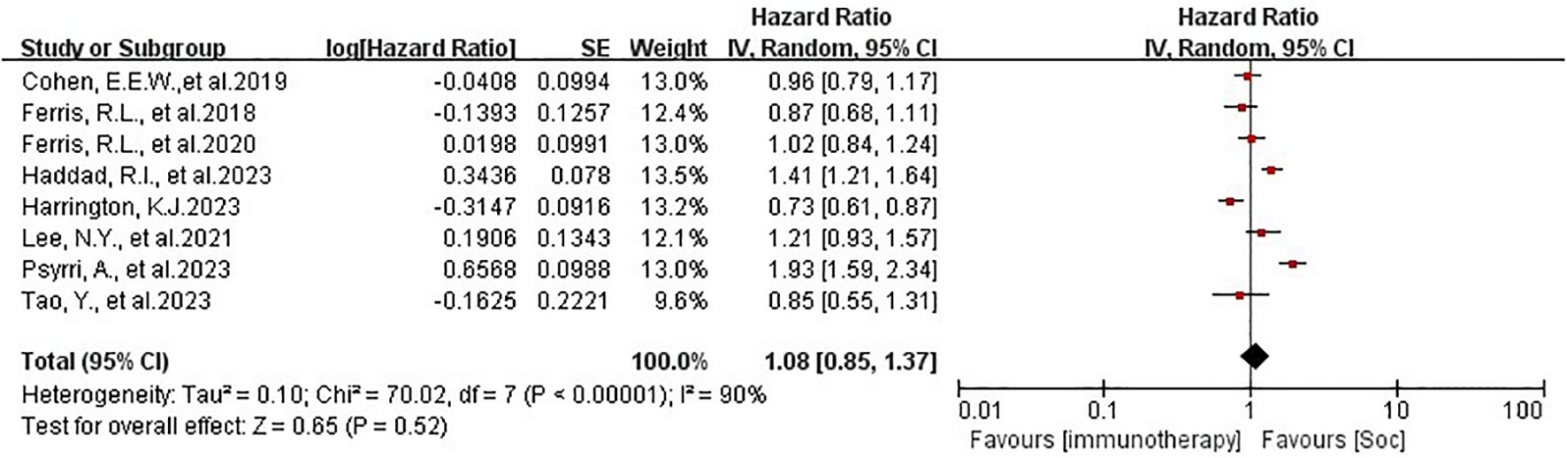
PFS analysis of immunotherapy in HNSCC. PFS, progression-free survival.

**Figure 4:**
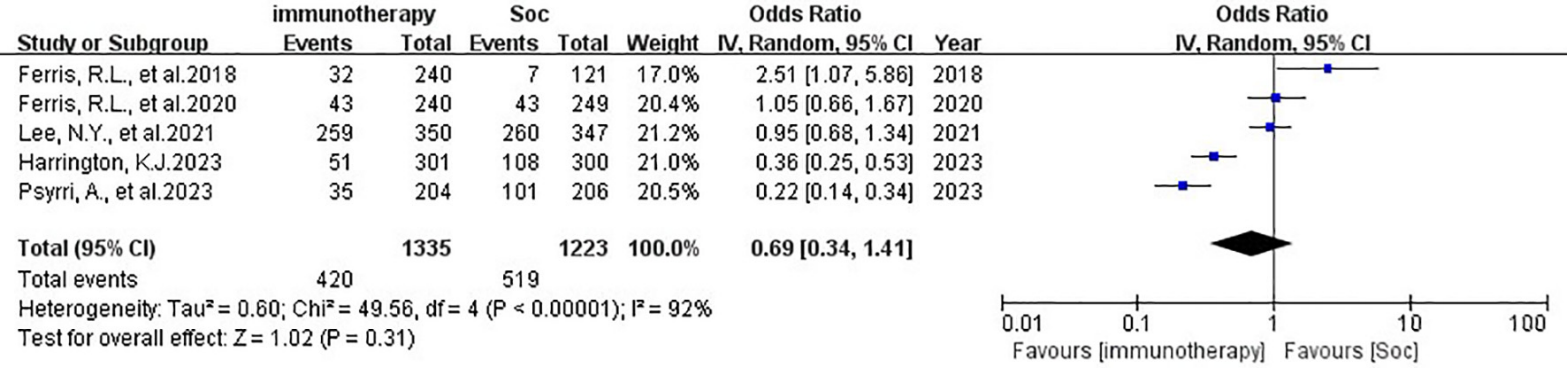
ORR analysis of immunotherapy in HNSCC. ORR, objective response rate.

**Figure 5:**
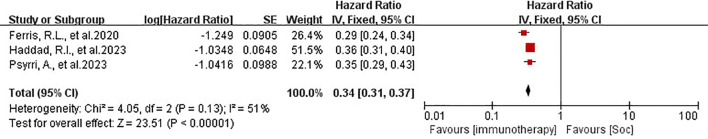
DoR analysis of immunotherapy in HNSCC. DoR, duration of response.

The patients in all trials were combined for the meta-analysis of OS demonstrated significant differences between experiment groups and control groups (HR 0.86, 95% CI 0.77-0.98, P=0.02, **Figures 2A, B**. Based on PFS, the result indicated that no significant efficacy difference between immunotherapy and non-immunotherapy (HR 1.08, 95% CI 0.85-1.37, P=0.52, **Figure 3**). For ORR, five articles with 2558 patients were analyzed, the immunotherapy group showed better response than Soc (OR 0.69, 95% CI 0.34-1.41, P=0.31, **Figure 4**). Besides, three trials (14, 16, 18) with a total of 2037 patients were included in the DoR analysis. A trend towards a significantly increased DoR in experimental groups than in control groups (HR 0.34, 95% CI 0.31-0.37, P<0.01, **Figure 5**

**Figure 6:**
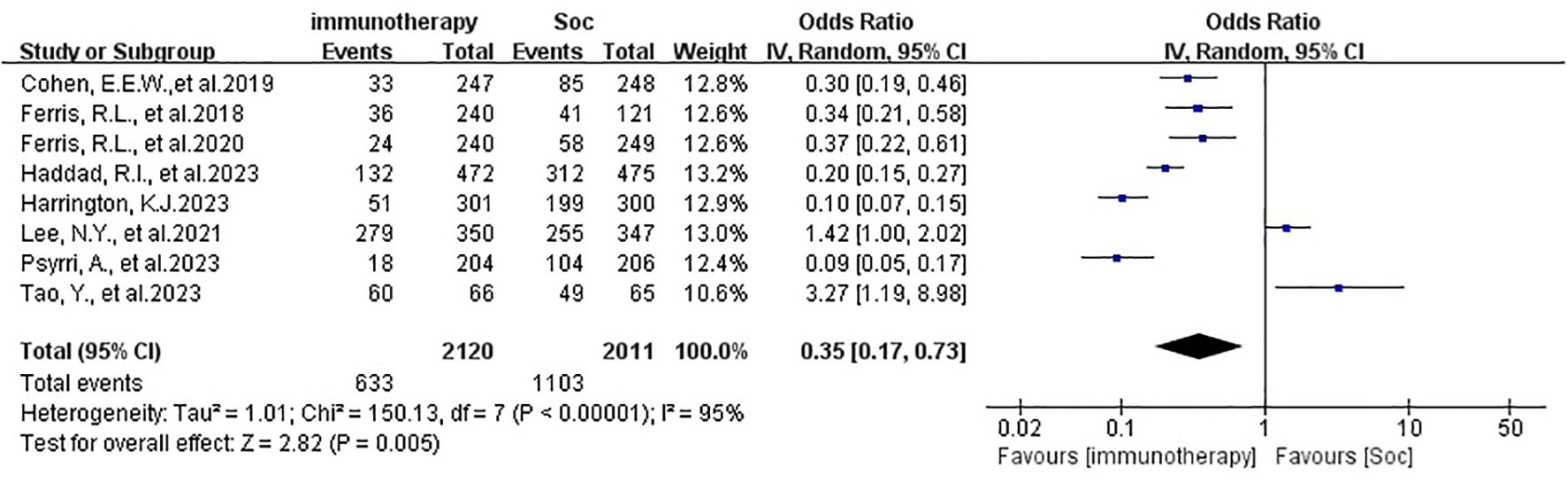
Grade 3-4 adverse reaction rate of immunotherapy in HNSCC.

In this meta-analysis, grade 3-4 treatment-related adverse events were administered with an acceptable safety profile in immunotherapy groups, supported by eight included studies (OR 0.35, 95% CI 0.17-0.73, P=0.01, **Figure 6**). The incidence of grade 3-4 TRAEs was 30.42% vs 54.65% compared with experiment groups and control groups. The most common > 3-grade treatment-related events of immunotherapy were fatigue, anemia, diarrhea, and skin reactions like rash (7, 16, 18). The most severe immune-related adverse events (irAE) were hepatitis, myocarditis, and Sjogren syndrome. All analyzed data are shown in **Table 2**. Prior similar meta-analysis outcomes are shown in **Supplementary Table 1**.

**Table 2:** Summary of the meta-analysis outcomes.

outcome	Number of studies	Number of patients	Effect estimate (95% CI)	P-value	Heterogeneity(I², p-value)
OS	8	4126	0.86 (0.77, 0.98)	0.02	52% (0.04)
PFS	8	4126	1.08 (0.85, 1.37)	0.52	90% (<0.01)
ORR	5	2558	0.69 (0.34, 1.41)	0.31	92% (<0.01)
DoR	3	2037	0.34 (0.31, 0.37)	<0.01	51% (0.13)
TRAEs	8	4131	0.35 (0.17, 0.73)	<0.01	95% (0.01)

## 4 Discussion

The therapeutic arsenal of HNSCC is rapidly evolving because of the introduction of new immunotherapeutic agents, which have been shown to improve treatment outcomes and OS in recurrent and metastatic disease and local advanced HNSCC (R/M/LA-HNSCC) (21). Our meta-analysis confirms immunotherapy was effective in OS with the same benefits observed in PFS, which is consistent with the Paderno, A.et al study (22) but inconsistent with Chen, L. et al. report (23). It may need to subdivision PD-L1 expression and HPV status and other so-on subgroups to analyze the reason.

CheckMate 651 (ClinicalTrial.gov identifier: NCT02741570) showed no statistically significant differences in OS with nivolumab plus ipilimumab versus EXTREME (platinum, 5-fluorouracil, and cetuximab in all randomly assigned R/M HNSCC (HR 0.95, 97.9% CI 0.80-1.13, P=0.4951), this study manifested no impact of median OS from the different primary site including oral cavity, oropharynx, hypopharynx, and larynx (HR 0.99, 95% CI 0.87-1.12, P=0.85) (16). JAVELIN Head and Neck 100 (ClinicalTrials.gov identifier: NCT02952586) informed avelumab was not prolonging PFS in patients with locally advanced squamous cell carcinoma of the head and neck (HR 1.21, 95% CI 0.93-1.57, P=0.92). Moreover, the subgroup of the primary site was generally consistent with the primary outcome in PFS (HR 1.14, 95% CI 0.87-1.48, P=0.34) (17).

KESTREL (ClinicalTrial.gov identifier: NCT02551159) and EAGLE (ClinicalTrial.gov identifier: NCT02369874) confirmed durvalumab plus tremelimumab was not superior to EXTREME or SoC (cetuximab, a taxane, methotrexate or a fluoropyrimidine) (14, 18). This means an addition of CTLA-4 inhibitors doesn’t improve either OS or PFS compared with PD-L1 inhibitor monotherapy in patients with R/M HNSCC. A meta-analysis supported this conclusion that a combination of durvalumab plus tremelimumab may achieve comparable outcomes in terms of ORR, OS, PFS, and DoR (24). KEYNOTE-048 (ClinicalTrial.gov identifier: NCT02358031) verified pembrolizumab-chemotherapy improved OS (HR 0.71, 95% CI 0.59-0.85) and PFS (HR 0.73, 95% CI 0.61-0.88) (15). A meta-analysis figured out that combining chemotherapy and PD-1 inhibitors in neoadjuvant treatment could improve ORR compared with immunotherapy only (61% vs 22%) (25). Patil, V. M. et al. reported that low-dose nivolumab plus triple metronomic chemotherapy (TMC-I) improved OS more than TMC in recurrent or newly diagnosed advanced HNSCC (HR 0.545, 95% CI 0.36-0.82) (26).

This study had several limitations. First, the objective response rate was not investigated due to the high heterogeneity of data. Second, there was substantial diversity in the included studies in terms of the treatment regimens and agents. This clinical heterogeneity could be considered a potential problem in interpreting the results of the present meta-analysis. Third, the included RCTs are most of the open-label designs and were supported by pharmaceutical industry funding. Fourth, the efficacy biomarker of immunotherapy in HNSCC is still not unique, such as combined positive score (CPS) expression and human papillomavirus (HPV) status not enough to indicate the prognosis. Finally, long-term clinical outcomes like 5-year OS have not been evaluated.

## 5 Conclusion

To the best of our knowledge, this is the largest inclusion of HNSCC patients study of meta-analysis and the first focus on the efficacy of CTLA-4 immunotherapy in HNSCC review. This meta-analysis indicated that HNSCC patients could get clinical benefits from immunotherapy, either R/M HNSCC or LA HNSCC, especially PD-1 inhibitors. However, its routine use is hindered by its expense and the challenge of selecting patients who will truly benefit. More convincing results need more immunotherapy-applied studies involved. The excellent treatment protocol of HNSCC needs more long-term clinical data. In the future, we may try to explore the efficacy of neoadjuvant low-dose immuno-chemotherapy in LA-HNSCC and attempt to define the boundaries of tumor reduction surgery results in protecting more organ functions.

## Data Availability

The original contributions presented in the study are included in the article/**Supplementary Material**, further inquiries can be directed to the corresponding author/s.
