# Supplementary figures and images for: Efficacy and safety of immunotherapy for head and neck squamous cell carcinoma: a meta-analysis of randomized clinical trials
Source: Front Oncol. 2025 Jan 9;14:1489451. doi: 10.3389/fonc.2024.1489451 (PMC11755100; doi:10.3389/fonc.2024.1489451)

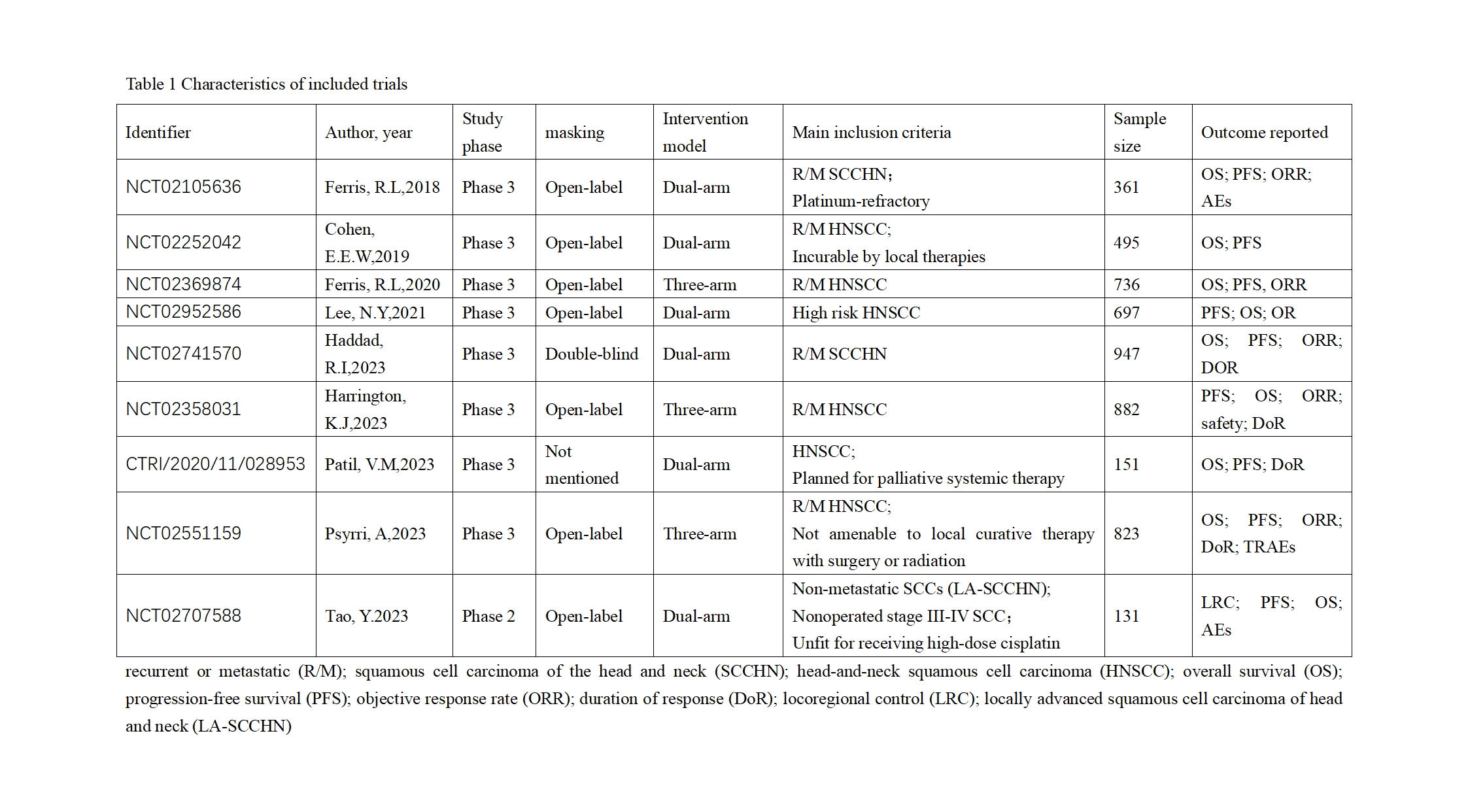

Supplement: Supplementary file 1 [file Image1.tif]

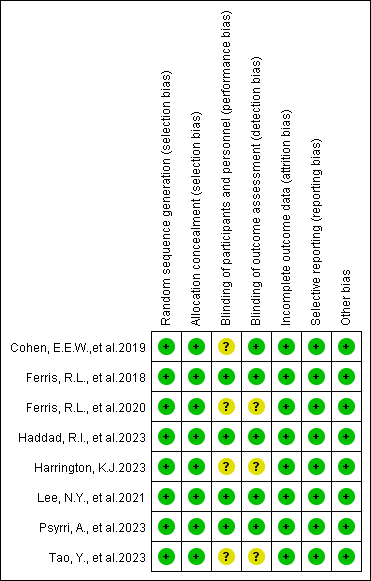

Supplement: Supplementary file 2 [file Image2.png]
