# Supplementary material for: Efficacy and safety of immunotherapy for head and neck squamous cell carcinoma: a meta-analysis of randomized clinical trials
Source: Front Oncol. 2025 Jan 9;14:1489451. doi: 10.3389/fonc.2024.1489451 (PMC11755100; doi:10.3389/fonc.2024.1489451)
Supplement: Supplementary file 4 [file Table1.docx]

Supplement Table 1 A summary of similar prior meta-analysis

| Author, year | Title | Included studies | Patients (number) | OS  (HR, 95% CI) | PFS  (HR, 95% CI) | ORR  (OR, 95% CI) | >3G TRAEs  (OR, 95% CI) |
| --- | --- | --- | --- | --- | --- | --- | --- |
| Paderno,A.,2024 | The predictive role of PD-L1 in head and neck cancer:  A systematic review and meta-analysis | 7 | R/M HNSCC  (n=4477) | \| 0.80  (0.70, 0.92) \| \| --- \| | \| 1.04  (0.83,1.30) \| \| --- \| | NA | NA |
| Liu, C, 2024 | Evaluating the efficacy and safety of different neoadjuvant  immunotherapy combinations in locally advanced HNSCC:  a systematic review and  meta-analysis | 32 | LA HNSCC  （n=1943） | NA | NA | 0.67  (0.57, 0.78) | 0.35  (0.20, 0.50) |
| Han, X, 2023 | Durvalumab with or without tremelimumab for patients with recurrent or metastatic squamous cell carcinoma of the head and neck:a systematic review and meta-analysis | 3 | R/M HNSCC  (n=1298) | NA | NA | 1.15  (0.85, 1.56) | 1.93  (1.36, 2.73) |
| Chen, L, 2023 | Combination therapy with immune checkpoint inhibitors in recurrent or metastatic squamous cell carcinoma of the head and neck:A meta-analysis | 5 | R/M HNSCC  (n=2576) | 0.73 (0.62, 0.87) | 0.65  (0.43, 0.99) | 1.10  (1.01, 1.19) | 0.79  (0.56, 1.11) |
| Zhang,S, 2022 | Efficacy of cetuximab plus PD-1 inhibitor differs by HPV status in head and neck squamous cell carcinoma: a systematic review and meta-analysis | 7 | R/M HNSCC  (n=802) | 1.37 (0.80, 2.36) | NA | 1.19  (0.51, 2.74) | NA |
